# A Robust High Throughput Platform to Generate Functional Recombinant Monoclonal Antibodies Using Rabbit B Cells from Peripheral Blood

**DOI:** 10.1371/journal.pone.0086184

**Published:** 2014-02-04

**Authors:** Stefan Seeber, Francesca Ros, Irmgard Thorey, Georg Tiefenthaler, Klaus Kaluza, Valeria Lifke, Jens André Alexander Fischer, Stefan Klostermann, Josef Endl, Erhard Kopetzki, Achal Pashine, Basile Siewe, Brigitte Kaluza, Josef Platzer, Sonja Offner

**Affiliations:** Large Molecule Research, Pharma Research and Early Development, Roche Diagnostics GmbH, Penzberg, Germany; Simon Fraser University, Canada

## Abstract

We have developed a robust platform to generate and functionally characterize rabbit-derived antibodies using B cells from peripheral blood. The rapid high throughput procedure generates a diverse set of antibodies, yet requires only few animals to be immunized without the need to sacrifice them. The workflow includes (i) the identification and isolation of single B cells from rabbit blood expressing IgG antibodies, (ii) an elaborate short term B-cell cultivation to produce sufficient monoclonal antigen specific IgG for comprehensive phenotype screens, (iii) the isolation of VH and VL coding regions via PCR from B-cell clones producing antigen specific and functional antibodies followed by the sequence determination, and (iv) the recombinant expression and purification of IgG antibodies. The fully integrated and to a large degree automated platform (demonstrated in this paper using IL1RL1 immunized rabbits) yielded clonal and very diverse IL1RL1-specific and functional IL1RL1-inhibiting rabbit antibodies. These functional IgGs from individual animals were obtained at a short time range after immunization and could be identified already during primary screening, thus substantially lowering the workload for the subsequent B-cell PCR workflow. Early availability of sequence information permits one to select early-on function- and sequence-diverse antibodies for further characterization. In summary, this powerful technology platform has proven to be an efficient and robust method for the rapid generation of antigen specific and functional monoclonal rabbit antibodies without sacrificing the immunized animal.

## Introduction

Rabbit antibodies have a proven track record for the use in *in vitro* diagnostics, since they combine high affinity with high specificity even towards antigens that are weakly immunogenic in mice. Furthermore, antibodies that are cross-reactive with the respective murine orthologs are more frequently produced in rabbits than in mice due to immunological tolerance (reviewed in [1]). These specific features of rabbit antibodies are not only highly preferred for diagnostic antibodies but also for therapeutic antibodies. Especially the cross-reactivity to the respective murine protein counterpart opens up the possibility to use these antibodies in mouse models of human disease.

For both therapeutic and diagnostics applications, monoclonal antibodies are more suitable than polyclonal antibodies. Currently, the standard procedures to produce rabbit monoclonal antibodies are either by hybridoma generation using a specific rabbit fusion cell line [2] or by phage display using rabbit spleen as a source for the variable (V) regions of the heavy (VH) and light (VL) chains [3], [4]. However, rabbit hybridomas were found to be less stable than conventional mouse or rat hybridomas [5, and confirmed by our own observations (unpublished data)]. In addition, the hybridoma generation as well as the phage display approach using the spleen of an immunized rabbit as a source of antigen specific B cells allow only a single sampling point at the end of the immunization period and require the sacrifice of the animal [4].

Pioneering work in the B-cell field encompassed the generation of the feeder cell line “EL-4 B5” which in combination with a specific cytokine mixture enables the cultivation of murine and human immunoglobulin (Ig) secreting B-cell clones [6] consisting of antibody-secreting cells (ASCs) or plasma cells. To date, several adaptations of this protocol as well as completely new technologies using advanced PCR-based methods are available for sampling and characterizing antigen specific B cells from spleen and from blood of immunized animals. However, these technologies require extensive expression cloning efforts to obtain a reasonable number of antigen specific and functional monoclonal antibodies mainly for two reasons: (i) the IgG amount in the supernatant is so low that only one or two binding assays can be performed excluding functional assays, resulting at best in a plethora of antigen binding supernatants [7]–[15], or (ii) the cultivation of a pool of different lymphocytes including polyclonal antigen specific B cells requires that each of the possible heavy (HC) and light chain (LC) pairs has to be cloned and characterized separately [16], [17].

Our goal was to overcome the above mentioned limitations by providing a robust high throughput technology for the production of monoclonal and antigen specific rabbit antibodies that are particularly enriched for functional antibodies. Therefore, it was necessary to establish the handling, the sorting and the cultivation of primary (non-immortalized) rabbit B cells, as well as the V region amplification using the polymerase chain reaction (PCR) and the subsequent expression cloning workflow in such a way that (i) the peripheral blood as a source for the antigen specific B cells could be used allowing a faster sampling schedule, consecutive sampling points in time, and the survival of the immunized animals, (ii) a B-cell selection step was introduced enabling the enrichment of antigen specific peripheral B cells, (iii) the supernatant of the rabbit B-cell clones (ASCs) contains sufficient monoclonal IgG to enable extensive screening and to unambiguously identify antigen specific and functional rabbit antibodies prior to the V-region PCR amplification, and (iv) a robust PCR and expression cloning workflow ensures a high overall yield. Rabbits are especially suited for this technology since (i) their body size allows blood samples with a sufficient volume for isolating antigen specific peripheral B cells, (ii) they are outbred and therefore deliver an animal specific B-cell repertoire, and (iii) they are easier to house than guinea pigs, sheep or goats.

Accordingly, rabbits immunized with the human IL1RL1 antigen (also known as ST2, DER4, FIT-1, IL33R, ST2L, ST2V, T1) were used for the proof-of-concept study. The IL33-IL1RL1 ligand-receptor system plays an important role in diseases such as asthma [18], [19], ulcerative colitis [20] or arthritis [21]. IL-33 was described as a member of the IL-1 family of cytokines [22] and as a ligand for the orphan IL-1R family receptor IL1RL1 [23]. IL1RL1 together with the IL-1 receptor accessory protein form the IL-33 receptor complex [24]. The treatment of mice with a soluble IL1RL1-Ig Fc fusion protein as decoy receptor or with a blocking anti-IL1RL1 antibody resulted in a reduction of the collagen induced arthritis [21] and of the inflammatory response in the lung [25] proving the therapeutic potential of the IL-33 and IL1RL1 interaction. Therefore, the ideal therapeutic antibody should fulfill two functional criteria: blocking the binding of the human IL-33 ligand to IL1RL1 to prevent pathogenesis, and being cross-reactive to the cynomolgus and murine protein orthologs for functional studies in these species. In addition, for therapy of human diseases a humanization of the functional rabbit antibody is required to reduce possible immunogenicity issues in the clinic.

We describe here for the first time a protocol for the efficient generation of antigen specific and functional monoclonal rabbit antibodies from single peripheral rabbit B cells using an elaborate B-cell cloning process and a robust B-cell PCR workflow.

## Materials and Methods

### Antigen Supply

Antigens for immunization and screening were generated by gene synthesis and recombinant expression in HEK293 cells also known as HEK293F cells (Life Technologies). For expression of soluble human IL1RL1 the sequence was taken from EMBL em:d12763 (E78 variant; isoform B 324–328 (splice variant; IDHHS to SKECF). For expression of soluble murine IL1RL1 the sequence was taken from Swissprot SW:ILRL1_MOUSE. For expression of soluble cynomolgus IL1RL1 the sequence was taken from full-length cynomolgus IL1RL1 derived from cynomolgus stomach cDNA (DEQ-3 variant, cloned and sequenced at Roche; unpublished data). All soluble IL1RL1-receptor molecules were expressed as N-terminal fusion proteins with a human IgG1 Fc constant region using the human CD33-signal peptide and under the control of the CMV-promoter. The transfection of HEK293 cells was performed as described below but in larger volumes of 500–1000 ml. After one week the Fc fusion proteins were purified from filtrated cell culture supernatants. In brief, the Fc fusion proteins were purified by Protein A affinity chromatography (Sepharose column, GE Healthcare). After washing with PBS the Fc fusion proteins were eluted at acidic pH and the Fc fusion protein containing fractions were immediately neutralized. Aggregated protein was separated from monomeric fusion proteins by size exclusion chromatography (Superdex 200, GE Healthcare) in 20 mM Histidine, 140 mM NaCl pH 6.0. Monomeric Fc fusion protein fractions were pooled, concentrated if required using e.g. a MILLIPORE Amicon Ultra (30KD MWCO) centrifugal concentrator and stored at −80°C. Sample aliquots were used for subsequent analytical characterization e.g. by SDS-PAGE, size exclusion chromatography, mass spectrometry and endotoxin determination.

### Immunization

New Zealand White (NZW) Rabbits obtained from Charles River Laboratories International, Inc. were used for immunization. The rabbits were housed according to the Appendix A “Guidelines for accommodation and care of animals” in an AAALACi (Association for the Assessment and Accreditation of Laboratory Animal Care international) accredited animal facility. All animal immunization protocols and experiments were approved by the Government of Upper Bavaria (permit number 55.2-1-54-2531-76-09) and performed according to the German Animal Welfare Act and the Directive 2010/63 of the European Parliament and Council. Purified, HEK293-derived human IL1RL1 fused to the Fc region of human IgG1 was dissolved in NaCl-Histidin buffer pH 6.1 and mixed with an equal volume of complete Freund´s adjuvant (CFA) till generation of a stable emulsion. We immunized 3 rabbits (animal 68, 69, and 70) following an in-house immunization protocol which has been established and used at Roche Diagnostics for the generation of antisera including antisera against weakly immunogenic antigens or mammalian antigens that are highly conserved between species. For the implementation of the various steps of the B-cell cloning process and in order to avoid the introduction of additional variables, we decided to use this immunization protocol. The immunization protocol includes the repeated injection of immunogen emulsified with CFA into the same animal rotating different application routes (Table 1). Titers were monitored regularly during the immunization period. From each animal samples of 10 ml peripheral whole blood were collected 4–6 days after the third, fourth, fifth and sixth injection by ear bleed, respectively, and used for B-cell isolation (Table 1 and below).

**Table 1:** Immunization and bleeding protocol.

Immunization	Day	Immunogen	Application Route	Blood Samples
1	0	400 µg ILRL1-huFc in NaCl-Histidin buffer/CFA, 1∶1 mixture	Intradermal (multiple sites)	
2	7	200 µg ILRL1-huFc in NaCl-Histidin buffer/CFA, 1∶1 mixture	Intramuscular (i.m.)	
3	14	200 µg ILRL1-huFc in NaCl-Histidin buffer/CFA, 1∶1 mixture	Subcutaneous (s.c.)	1 Bleed per animal, either 4, 5 or 6 days after immunization
4	28	200 µg ILRL1-huFc in NaCl-Histidin buffer/CFA, 1∶1 mixture	i.m.	1 Bleed per animal, either 4, 5 or 6 days after immunization
5	56	200 µg ILRL1-huFc in NaCl-Histidin buffer/CFA, 1∶1 mixture	s.c.	1 Bleed per animal, either 4, 5 or 6 days after immunization
6	91	200 µg ILRL1-huFc in NaCl-Histidin buffer/CFA, 1∶1 mixture	i.m.	1 Bleed per animal, either 4, 5 or 6 days after immunization

### Antibody Titer

The antibody response to the immunization was determined by an IL1RL1 specific ELISA using serially diluted sera. 96-well MaxiSorp microtiter plates were coated with 0.3 µg/ml human IL1RL1 protein in 50 mM carbonate buffer, pH 9.55 for 1 h at 37°C. Thereafter the wells were blocked with PBS supplemented with 1% Crotein C (Roche Diagnostics GmbH) overnight at 4°C. After incubation with the appropriate serum goat anti-rabbit IgG linked to horseradish peroxidase (HRP, Jackson ImmunoResearch) was used for detection at a 1∶16000 dilution. BM blue^®^ HRP substrate solution (BM blue^®^: 3, 3′-5, 5′-Tetramethylbenzidine, Roche Diagnostics GmbH) was used for visualization. Reaction was stopped with 1 N HCl and measured by absorbance at 450/690 nm. We performed a functional characterization of the polyclonal sera of the IL1RL1-immunized rabbit using the cellular assay but unfortunately even the pre-immune sera produced a very high background in this assay. It was not possible to use this assay for the discrimination of the rabbits (data not shown). In addition, we could not exclude the presence of both antagonistic and agonistic antibodies in the polyclonal serum blurring the result of the functional assay. Therefore, we exclusively assessed the monoclonal antibodies and not the polyclonal sera in the functional biochemical and cellular assays.

### Rabbit B-cell Isolation

For the isolation of peripheral blood mononuclear cells (PBMCs) EDTA containing blood samples of rabbits were diluted twofold with 1x PBS before density centrifugation on Lympholyte M® (Cedarlane Laboratories). PBMCs were washed twice before staining with antibodies. Sterile 6-well plates (cell culture grade) were used to deplete macrophages and monocytes through unspecific adhesion. Each well was filled at maximum with 4 ml media containing up to 6×10^6^ PBMCs from the immunized rabbit. The cell suspension was allowed to bind for 1 h at 37°C in 5% CO_2_. Of the total cells in the supernatant, 90% were used for the panning step; the remaining 10% of cells were kept on ice until the immune fluorescence staining. Sterile cell culture 6-well plates were coated with 2 µg/ml human IL1RL1 protein in carbonate buffer (0.1 M sodium bicarbonate, 34 mM disodiumhydrogencarbonate, pH 9.55) overnight at 4°C. Plates were washed in sterile PBS 3x before use. 6-well tissue culture plates coated with human IL1RL1 protein were seeded with up to 6×10^6^ cells per 4 ml medium and the cells were incubated for 1 h at 37°C in 5% CO_2_. After the enrichment step on human IL1RL1 protein (panning) non-adherent cells were removed by carefully washing the wells 1–2x with 1x PBS. The remaining sticky cells were detached by trypsin (PAN Biotech) for 10 min at 37°C in 5% CO_2_ and then washed twice in media. The cells were kept on ice until the immune fluorescence staining.

### Immune Fluorescence Staining and Flow Cytometry

The FITC labeled anti-rabbit IgG used for single cell sorting was from AbD Serotec (Düsseldorf, Germany). For surface staining, cells from the macrophage depletion and/or antigen panning step were incubated with the optimally diluted anti-rabbit IgG FITC antibody in PBS for 30 min at 4°C in the dark. Following centrifugation, the supernatants were removed by aspiration. The PBMCs were subjected to two cycles of centrifugation and washing with ice-cold PBS. Finally the PBMCs were resuspended in ice-cold PBS and immediately subjected to the FACS analyses. Propidium iodide in a concentration of 5 µg/ml (BD Biosciences) was added prior to the FACS analyses to discriminate between dead and live cells. A Becton Dickinson FACSAria equipped with a computer and the FACSDiva software (BD Biosciences) were used for single cell sorting.

### Media

Standard medium was prepared with RPMI 1640 (PAN Biotech) and supplemented with 10% FCS (Hyclone), 2 mM Glutamine, 1% penicillin/streptomycin solution (PAA), 2 mM sodium pyruvate, 10 mM HEPES (PAN Biotech) and 0.05 mM 2-mercaptoethanole (Gibco). The Pansorbin Cells (i.e. *Staphylococcus aureus* strain Cowans (SAC); Merck Millipore) were used in an 1∶20000 dilution.

### Preparation of a Rabbit Thymocyte Supernatant (TSN)

Rabbit T cells and rabbit macrophages were used to generate rabbit specific cytokines (rabbit TSN) according to the generation of human and murine TSN [17], [26] with the modification that the rabbit T-cell precursors were isolated from the thymus of 4–5 week-old rabbits. These thymocytes were centrifuged and immediately cultivated or frozen in aliquots of 3×10^7^ cells. For cultivation, the thymocytes were seeded with a cell density of 5×10^5^ cells/ml at minimum in cell culture flasks using standard medium and incubated for 48 h at 37°C. PBMCs isolated from blood of adult rabbits using Lympholyte M® were used to enrich monocytes/macrophages by adherence at 37°C in standard medium at a minimum cell density of 1×10^6^ cells/ml, usually in 175 cm^2^ cell culture flasks at a volume of 35–40 ml per flask. After 1–1.5 h, non-adherent cells were removed by washing with fresh warm standard medium. Attached monocytes/macrophages were cultivated for 48 h in standard medium. T cells and macrophages obtained from different rabbits were kept in separate flasks. Prior to the mixing of T cells and macrophages, T cells were centrifuged (800×g for 10 min) and resuspended in fresh standard medium containing 10 ng/ml phorbol-12-myristate-13-acetate (PMA; Sigma-Aldrich) and 5 µg/ml phytohemagglutinin M (PHA-M; Sigma-Aldrich) at a minimal cell density of 5×10^5^ cells/ml. The medium was removed from the macrophage cultures and replaced by the T-cell suspension (35–40 ml per 175 cm^2^ cell culture flask). After 36 h of co-cultivation, the T cell/macrophage conditioned medium was removed and termed “rabbit TSN” (rbTSN). For removal of remaining cells, the TSN solution was filtered through a 0.22 µm filter and stored frozen at −80°C.

### B-cell Cultivation

Single sorted rabbit B cells were cultivated in 96-well round bottom plates with 210 µl/well standard medium containing 5% rabbit thymocyte supernatant (rbTSN, in-house production) and 2 × 10^4^ gamma-irradiated murine EL-4 B5 feeder cells per well [6] for 7 days at 37°C in 5% CO_2_. The B-cell cultivation supernatants were removed for screening. In general, the quality of the B-cell cloning process was assessed by the frequency of IgG secreting B-cell clones (% total wells or % IgG-positive wells), the average (avg) IgG concentration of all IgG-secreting B-cell clones and if applicable the frequency of B-cell clones secreting antigen specific monoclonal antibodies (% total wells or % IgG-positive wells). An IgG-secreting B-cell clone consists of clonally expanded ASCs/plasma cells. After the removal of the supernatant the B-cell clones were lyzed by 100 µl RLT buffer (Qiagen, Hilden, Germany) in the 96-well round bottom plates. The mRNA containing lysates were subsequently transferred to DNA LoBind 96-well deepwell plates (Eppendorf) and immediately frozen at −20°C for storage until the recovery of the variable gene regions starting with the mRNA isolation.

### mRNA Isolation and cDNA Synthesis

Total RNA of the ASC/feeder cell lysates was prepared using the Total RNA Isolation Kit NucleoSpin (Macherey & Nagel) according to the manufacturer's instructions. The cDNA was generated by reverse transcription of the mRNA using the Super Script III first-strand synthesis SuperMix (Invitrogen) according to the manufacturer's instructions. In a first step 6 µl of the isolated mRNA was mixed with 1 µl annealing buffer and 1 µl (50 µM) oligo dT, incubated for 5 min at 65°C and thereafter immediately placed on ice for about 1 min. Subsequently, while still on ice, 10 µl 2x First-Strand Reaction Mix and SuperScript™ III/RNaseOUT™ Enzyme Mix were added. After mixing the reaction was placed for 50 min at 50°C. The reaction was terminated by incubation at 85°C for 5 min. After termination the reaction mix was placed on ice.

### PCR of Cognate VH and VL Gene Segments

The PCR amplification of the V regions of rabbit B cells is facilitated by the fact that rabbits preferentially use the proximal (3′) VH gene segment in the IgH locus, VH1, for VDJ rearrangement and that they have only a single gene for the constant region of IgG [27]–[31]. The VH forward primer is specific for the leader sequences of the rabbit allotypes VH1a1 and VH1a3. The VH forward primer sequence is 5′AAGCTTGCCACCATGGAGACTGGGCTGCGCTGGCTTC. The reverse primer was placed within rabbit IgG CH1 region and has the sequence 5′CCATTGGTGAGGGTGCCCGAG. The VL forward primer is specific for 22 rabbit kappa VL region leader sequences, with one mixed codon usage (Y = C or T). In wild type rabbits more than 70% of antibody LCs are derived from the kappa1 locus, the remainder from kappa2 and lambda [32]. The V kappa forward primer sequence is 5′AAGCTTGCCACCATGGACAYGAGGGCCCCCACTC. The reverse primer was placed within the rabbit kappa constant region and has the sequence 5′CAGAGTRCTGCTGAGGTTGTAGGTAC (R =  G or A). A Kozak consensus sequence [33] was included in the 5′ region of the forward primers. The PCR was carried out using AccuPrime Pfx SuperMix (Invitrogen) according to the manufacturer’s instructions. The VL and VH regions were amplified in separate reactions. PCR-primers (0.2 µM per reaction) with 25 bp overlaps to target antibody expression vectors were used. After the PCR 8 µl of the PCR reaction mixture were used for analysis on 48-well eGels (Invitrogen). Residual PCR primers were removed using the NucleoSpin® 96 Extract II kit (Macherey & Nagel) according to the manufacturer’s instructions. The DNA sequences encoding the VHs and VLs were obtained by sequencing the PCR products, performed by SequiServe GmbH (Vatterstetten).

### Cloning and Recombinant Expression of Cognate VH and VL Gene Segments

The prototype cDNA expression plasmid used for the transient recombinant expression of the HC and LC of monoclonal rabbit antibodies contained an expression cassette consisting of a 5′ CMV promoter including intron A, and a 3′ BGH polyadenylation sequence (see Figures S1 A and B). In addition to the expression cassette, the plasmids contained a pUC18-derived origin of replication and a beta-lactamase gene conferring ampicillin resistance for plasmid amplification in *E. coli*. Further, two variants of the basic plasmid were used: one plasmid contained the rabbit IgG constant region designed to accept the VH regions while a second plasmid contained a generic rabbit kappa LC to accept the VL regions. For cloning of the respective amplification product, these plasmids were first linearized by restriction enzyme digestion. Subsequently, the linearized plasmid DNA was purified by preparative agarose electrophoresis and extracted from the gel (QIAquick Gel Extraction Kit, Qiagen). This purified and linearized plasmid DNA was added to a PCR-mix as template using primers overlapping by 20–25 bp with the PCR-products to be cloned. The PCR was carried out using AccuPrime Pfx SuperMix (Invitrogen). The PCR-products derived from the individual rabbit B-cells were cloned into the respective expression vectors using a “sequence and ligation independent cloning” (SLIC) method [34], [35]. The purified linear vector and the insert were treated with 0.5 U T4 DNA polymerase (Roche Applied Sciences, Mannheim, Germany) per 1 µg DNA for 45 min at 25°C in the absence of dNTPs to generate matching overhangs. The reaction was stopped by adding 1/10th of the reaction volume of a 10 mM dCTP solution (Invitrogen). The T4 treated vector and insert DNA fragments were combined at a plasmid:insert ratio of 1∶2 (w/w) (e.g. 100 ng:200 ng) and recombined by adding RecA protein (New England Biolabs) and 10x RecA Buffer for 30 min at 37°C. Subsequently, 5 µl of each of the generated HC and LC expression plasmids were used to transform MultiShot Strip Well TOP 10 chemically competent *E. coli* cells (Invitrogen) using a standard chemical transformation protocol. Briefly, the competent cells were thawed and kept at 4°C before adding 5 µl of the HC and LC expression plasmids. The transformation mix was kept at 4°C for another 10 min, was subsequently incubated at 42°C for exactly 30 sec and was finally cooled down at 4°C again for 2 min. After regeneration (shaking the transformed *E. coli* cells for 45 min at 37°C) the entire transformation mixture was transferred into DWP 96 (deep well plates) containing 2 ml of LB medium supplemented with ampicillin. The cells were incubated in a shaker for 20 h at 37°C. Next, the plasmid DNAs encoding the respective immunoglobulin HC and LC) constant regions were purified using the NucleoSpin 96 Plasmid Mini Kit (Macherey & Nagel), digested with selected restriction enzymes, and analyzed on 48-well eGels (Invitrogen). In parallel, glycerol stocks were prepared for storage. All pipetting steps of the workflow described above were performed using microtiter plates and an EpMotion laboratory robot (Eppendorf) to increase throughput and decrease error rates. Finally, the individual rabbit antibodies were recombinantly expressed in HEK293 cells. To this end, the HEK293 cells were grown in a shaking device at 120 rpm in F17-medium (Gibco) at 37°C in an atmosphere containing 8% CO_2_. Cells were split the day before transfection and seeded at a density of 0.7–0.8×10^6^ cells/ml. On the day of transfection, 1–1.5×10^6^ HEK293 cells in a volume of 2 ml were transfected with 0.5 µg HC plasmid plus 0.5 µg LC plasmid, suspended in 80 µl OptiMEM® medium (Gibco) and supplemented with 1 µl 293-free transfection reagent (Novagen) in 48-well deep well plates. Cultures were incubated for 7 days at 180 rpm at 37°C and in 8% CO_2_. After 7 days the culture supernatants were harvested, purified by Protein A column standard protocols and analyzed for antibody content and specificity.

### Sequence Analysis of the VH and VL Gene Region

Protein sequence data were analyzed by a specialized Excel-workbook. This tool takes a multiple sequence alignment for both the VH and the VL region as input and returns the following output results: (i) Sequence analysis: identification of the closest rabbit germ line (VH1a1, VH1a3), determination of the framework and the complementarity determining regions (CDRs), proposal of antibodies clustering according to sequence identity/similarity of both CDR-H3 and CDR-L3, calculation of characteristics of interest (e.g. CDR3 hydropathy, CDR length), (ii) Statistical analysis of the number of amino acid replacement mutations and determination of the mean mutation rate dependent on bleed, closest germ line and V-region (framework/CDR), (iii) Clone categorization according to bleed, animal, sort, closest germ line, epitope and sequence cluster (clones sharing identical or highly similar CDR3 sequences), identical VH/VL or identical CDR-H3/CDR-L3, and (vi) Dynamic visualization (sorting & grouping, coloring, filtering) of all sequences, hypermutation spots, sequence derived data and all screening assay data.

### Quantification of Rabbit IgG

A mixture of 0.5 µg/ml of biotinylated mouse anti-rabbit IgG antibody (Sigma-Aldrich) and 0.35 µg/ml anti-rabbit IgG HRP conjugate (Sigma-Aldrich) was transferred to 384 well streptavidin coated microtiter plates (MicroCoat Biotechnologie GmbH). Dilutions of B-cell supernatants in PBS supplemented with 0.5% BSA and 0.05% Tween-20 were added and incubated for 90 min at RT. After repeated washing (6x) with PBST (phosphate buffered saline with 0.2%Tween®-20) buffer the plates were developed with BM blue^®^ HRP substrate solution and color formation was measured by absorbance at 370 nm. A commercial rabbit IgG (Sigma-Aldrich) was used as a calibration standard.

### Antigen Binding Immunoassays

The test was performed at room temperature (RT) on 384-well streptavidin coated microtiter plates with PBS buffer supplemented with 0.05% Tween^®^-20 and 0.5% BSA (Roche Diagnostics GmbH). Biotinylated anti-human-Fc antibody (0.5 µg/ml, Jackson ImmunoResearch) was added to the wells containing 0.5 µg/ml human or murine IL1RL1 Fc fusion protein (R&D Systems) or cynomolgus IL1RL1 Fc fusion protein (in-house) with 1∶2000 diluted anti-rabbit Fc–HRP conjugate (GE Healthcare). Thereafter different dilutions of the B-cell supernatants were transferred to the wells. After 90 min incubation the plate was washed 6x with PBST and developed with BM blue^®^ HRP substrate solution for 30 min. Absorbance was measured at 370 nm. The blank value was defined without addition of antibody. For negative selection of antibodies binding to the human Fc tag of the IL1RL1 protein an immune assay was established with 0.25 µg/ml of biotinylated anti-human Fc antibody (Jackson ImmunoResearch) as capture antibody, 25 ng/ml of an in-house human IgG1 antibody as bait and 1∶2000 diluted anti-rabbit Fc-HRP conjugate.

### IL1RL1:IL33 Interaction Assay

384-well MaxiSorp microtiter plates (Sigma-Aldrich, Nunc) were coated with 1 µg/ml goat anti-human IgG Fc fragment (Jackson Immuno Research) for at least 2 h. Thereafter the wells were blocked with PBS supplemented with 0.1% Tween®-20 and 2% BSA for 1 h. Recombinant human IL1RL1 Fc fusion protein (60 ng/ml, R&D Systems) was captured on a plate for 1 h. Dilutions of supernatants from rabbit ASCs or purified antibodies in PBS with 0.5% BSA and 0.05% Tween®-20 were incubated with the receptor protein for 1 h. Biotinylated human IL33 (PeproTech) was added for an additional hour to build up the complex. IL33 was biotinylated with Sulfo-NHS-LC-Biotin (Thermo Scientific Pierce) according to the manufacturer’s protocol and purified using ZebaTM Desalt Spin Column (Thermo Scientific Pierce). Binding of the biotinylated IL33 to the complex was detected with 1∶4000 diluted streptavidin HRP (Roche Diagnostics GmbH). After 1 h the plates were washed 6x with PBST and developed with BM blue® HRP substrate solution for 12 min. Absorbance was measured at 370 nm. The negative control was defined without addition of IL1RL1 protein and the positive control was defined with all components but without antibody. IC50 values were fitted to a four-parameter equation using XLFit® software (IDBS, Surrey U.K.). At least three independent measurements were performed in triplicates.

### Epitope Grouping by Cross Competition ELISA

The anti-rabbit capture antibody (2 µg/ml, Jackson ImmunoResearch) was coated on MaxiSorb 384-well plate for 2 h. After washing 3x with PBST buffer the wells were blocked with PBS supplemented with 0.1% Tween®-20 and 2% BSA for 1 h. 150 ng/ml of the first monoclonal antibody were captured on a plate for 1 h followed by blocking of non-saturated sites with 50 µg/ml rabbit IgG (Jackson ImmunoResearch). The second rabbit antibody (375 ng/ml) were incubated on a separate polypropylene plate with 10 ng/ml of the antigen IL1RL1 Fc fusion protein (R&D Systems) for 1 h. The pre-incubated mixture of the second antibody and the antigen were then transferred to the plate containing the first antibody. After 1 h of incubation at RT and a washing step 1∶2000 diluted anti human-Fc-HRP conjugate (GE Healthcare) was added for 1 h. Plates were developed after 6x washing with BM blue^®^ HRP substrate solution and color formation was measured by absorbance at 370 nm.

### Functional NK Cell Assay

IL33 amplifies both T_H_1 and T_H_2 responses by activating different leukocytes, in particular NK cells [36]. Human PBMCs were isolated from healthy blood donors by Ficoll gradient. The blood sampling of healthy volunteers was approved by the local ethics committee (Bayerische Landesärztekammer, Munich) and subjects gave written, informed consent. The NK cells were purified from PBMC using the negative NK cell isolation kit (Miltenyi Biotec). The average purity was over 96%. For assessment of the IL1RL1-blocking functionality of the monoclonal rabbit antibodies, 1×10^5^ NK cells/well were pretreated with the rabbit antibodies or the respective isotype control antibodies at different concentrations, seeded into a 96-well flat bottom plate and incubated for 1 h at 37°C. NK cells were then stimulated with 10 ng/ml IL-33 (Peprotech) and 1 ng/ml IL-12 (Sigma-Aldrich) and incubated for 20 h in RPMI 1640 medium supplemented with 10% FCS, 1% sodium pyruvate and L-Glutamine, as well as 0.1% 2-Mercaptoethanol. After this, supernatants were harvested, centrifuged and tested for IFN-γ production. For IFN-γ quantification an in-house established ELISA or BD’s CBA flex set platform was used (human IFN-γ flex set, BD Biosciences) according to the manufacturer’s instructions using a FACS Canto II.

### Statistical Calculations

In order to assess similarities between different epitopes through epitope binning we applied a hierarchical clustering approach using Ward’s method to the data sets [37]. Hierarchical clustering allows for a manual selection of the number of clusters, that is the number of groups with similar epitopes. Initially each epitope forms a cluster of its own. In a step by step process different clusters are joined to bigger clusters based upon the Ward’s distance metric. Manual inspection of the resulting dendrogram and the bridged distances per cluster join let us to assume a total of 6 clusters. This selection could be confirmed by a visual inspection when we applied principal component analysis to the same data set and plotted the data in a three-dimensional scatterplot with the first three principal components as the axes. The same analyses on the transposed data sets (clustering the columns instead of the rows) revealed only minor differences. The calculation of the threshold (cut off) for the absorbance value (OD) of the antigen specific ELISA assays follows a three-step process. At first we perform an outlier analysis to remove all samples which obviously stick out. Afterwards we fit a Johnson Su distribution [38] to the remaining samples and finally calculate the 99% quantile of the distribution which we define as our threshold. Visual inspection confirms that our calculated threshold fits well to the region where there is a sharp kink in the graph of all sorted samples. All calculations were performed with the software JMP 10 (SAS Institute GmbH, Böblingen, Germany).

## Results

### B-Cell Cloning: High Throughput Isolation and Cultivation of Individual, Peripheral, and Antigen Specific Rabbit B cells

**Figure 1:**
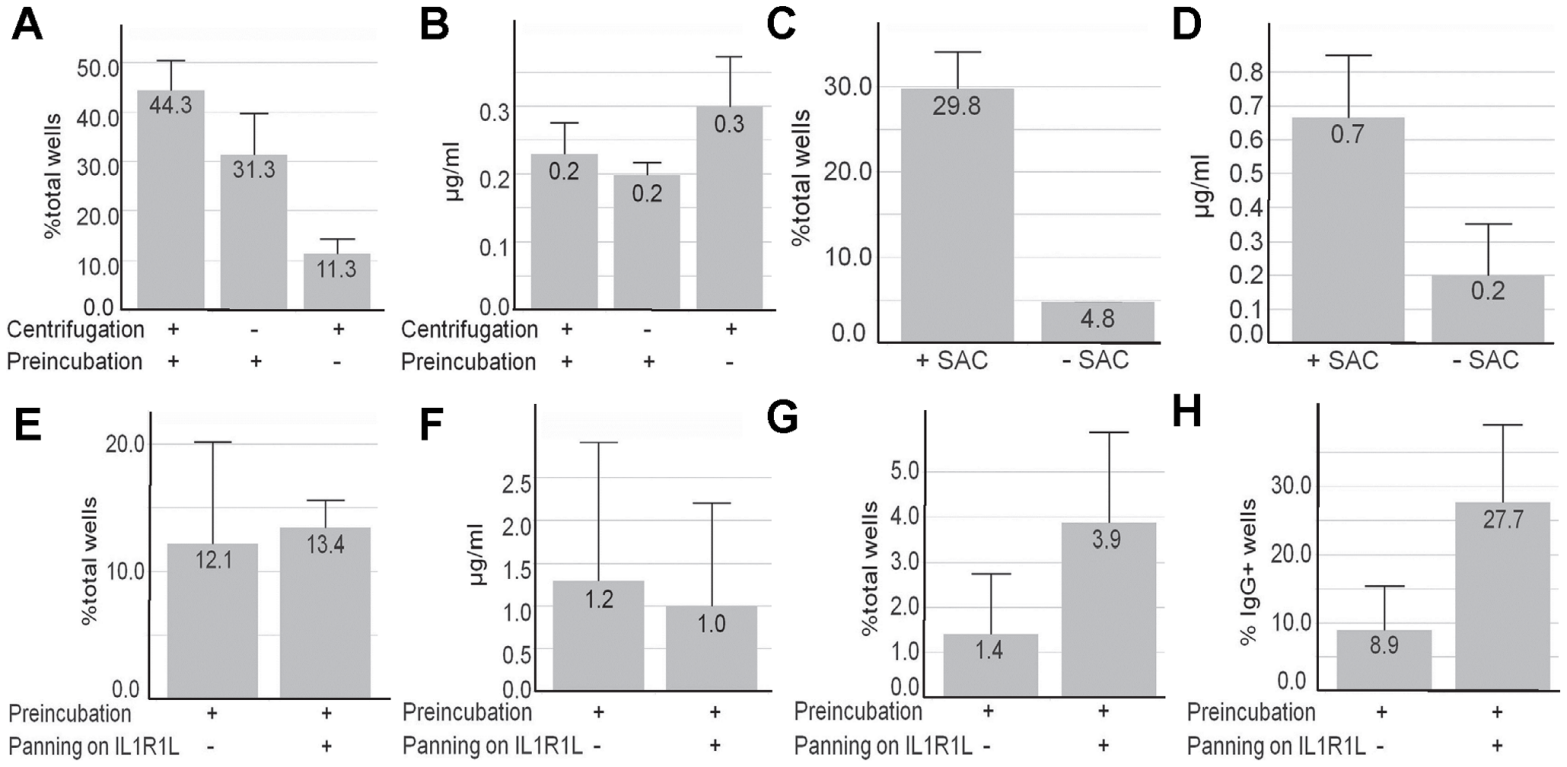
Influence of the improvements of the B-cell handling and the B-cell cultivation. (**A**) Percentage of IgG-producing B-cell clones per total wells and (**B**) average IgG concentration over all IgG-positive wells after +/− pre-incubation in medium and +/− centrifugation post sorting. For each parameter 368 wells were analyzed. (**C**) Percentage of IgG-producing B-cell clones (ASCs) per total wells and (**D**) average IgG concentration over all IgG-positive wells after using +/− SAC in a dilution of 1∶20000 during B-cell cultivation. For each parameter 252 wells were analyzed. (**E**) Percentage of IgG-producing B-cell clones per total wells, (**F**) average IgG concentration over all IgG-positive wells, as well as percentage of antigen specific B-cell clones (**G**) per total wells and (**H**) per IgG-producing B-cell clones after +/− protein panning. For each parameter around 3500 wells were analyzed. The error bars represent the standard deviation. The cut off value of IgG-positive wells was >0.013 µg/ml IgG and of human IL1RL1-positive wells was>OD 0.195.

It was of fundamental importance to grow and expand the non-immortalized, single sorted B cells derived from the blood of immunized rabbits in a robust and efficient manner. It proved to be beneficial to pre-incubate the freshly isolated PBMCs in standard medium for 1 h at 37°C prior to the staining of the cells and to bring the single sorted B cell into close contact to the EL-4 B5 feeder cells by centrifugation immediately post sorting. The combination of these two procedures allowed the generation of over 44% IgG-secreting B-cell clones (Figure 1A) consisting of ASCs/plasma cells. The frequency of the IgG secreting B-cell clones dropped by 25% without the centrifugation step or dropped fourfold without the pre-incubation step. However, the average (avg) IgG production was unaffected by both procedures (Figure 1B). The use of Pansorbin Cells (*S. aureus* strain Cowans, (SAC) [39]) as a supplement for the standard medium revealed that both, the average concentration of the secreted IgG as well as the frequency of IgG-secreting B-cell clones could be significantly increased after the cultivation period presumably by triggering toll-like receptors [40] (Figures 1C and D). In summary, the combination of the accelerated sedimentation, the pre-incubation in standard medium along with the TLR-activating supplement during the B-cell cultivation, maintained a high frequency of IgG secreting B-cell clones and a high average IgG concentration in the supernatants. The above mentioned results were obtained from B cells of unimmunized rabbits, but could reliably be transferred to B cells of immunized rabbits as described in the proof-of-concept study in this work.

The antigen specific peripheral B cells of immunized rabbits had to be enriched prior to the B-cell deposition in order to reduce the subsequent cultivation and screening efforts since FACS analyses of the rabbit PBMCs revealed that around 1–5×10^4^ IgG-positive B cells were present in a 10 ml blood sample (data not shown). This enrichment could be achieved by the specific adherence of the antigen specific surface IgG-positive B cells on antigen-coated plates (i.e. panning) [16], [26]. When using B cells from human IL1RL1-immunized rabbits (see below), the yield of IL1RL1-binding B-cell clones secreting IL1RL1-specific monoclonal antibodies was increased threefold by this enrichment step (Figures 1G and H). The frequency of IgG secreting B-cells (12% or 13% of the total wells) and the average IgG productivity (1.2 µg/ml or 1.0 µg/ml) was not influenced by the panning step (Figures 1E and F). Consequently, the panning on antigen was used as a standard procedure to enrich antigen specific B cells thereby increasing the efficiency of the whole workflow.

### B-Cell PCR: High Throughput Generation of Recombinant Rabbit Antibodies

The monoclonal rabbit antibodies were recombinantly produced using the mRNA of the B-cell lysates as template in order to yield a higher antibody concentration and to secure the genetic information. Each individual consecutive step of this workflow (mRNA isolation, cDNA synthesis, PCR amplification of the V regions, cloning into expression vectors and production of monoclonal rabbit antibodies) was analyzed and simplified to improve its efficiency and robustness with the primary objective of automation.

Accordingly, in a first step we determined the threshold for the mRNA isolation based on the IgG concentration of the primary B-cell supernatant by correlating the yield of the V-region PCR with the IgG concentration of the same sample. The mRNAs from 75 B-cell clones producing a broad range of IgG concentrations (0.001 µg/ml to 1.5 µg/ml) were isolated and used for reverse transcription (RT)-PCR. The comparison of the IgG concentrations of the individual supernatants with the respective PCR yield revealed that 85% of the B-cell clones allowed an efficient PCR amplification of the cognate VH and VL regions, if the respective supernatant contained more than 0.02 µg/ml IgG. Thus, we decided to define the concentration of 0.02 µg/ml rabbit IgG in the supernatant as the lower limit for selecting individual B-cell clones for expression cloning.

Furthermore, we analyzed whether we could process the nucleic acids from each individual B-cell clone as a pool starting with the isolation of mRNAs and ending with the final expression plasmids while avoiding the laborious isolation of single *E. coli* colonies. Therefore, *E. coli* cells transformed with plasmids containing the cognate VH/VL sequence pool of one B-cell clone were split into two aliquots. One aliquot was conventionally plated and single-clones picked before cultivation and the other aliquot was cultivated as a pool. The comparison of the VH and VL sequences of the isolated plasmids from the differently treated *E. coli* cells revealed that between 80% and 100% of the single colony-derived plasmids were identical to the sequences obtained from the corresponding pool-transformations (Table 2). Therefore, without losing the accuracy of the sequence information, the VH and VL encoding nucleic acid fragments derived from a single B-cell clone could be processed as a pool using only one single tube/well during the entire molecular biology workflow.

**Table 2:** Concordance of VL and VH sequences of single *E. coli* colonies derived from pool-cloning shown for 8 different B-cell clones (ASCs).

No. of B-cell clone	Number of the most abundant VL sequencesper total number of sequences	Number of the most abundant VH sequencesper total number of sequences
*5*	10/11	8/12
*6*	11/12	12/12
*10*	2/2	8/12
*35*	6/6	7/12
*38*	11/12	12/12
*39*	9/11	10/12
*42*	3/5	11/12
*50*	6/6	6/7

Finally, a ligation-independent cloning system [34], [35] was adapted to the requirements for the B-Cell PCR workflow. A comparison of the classical and the ligation-independent cloning demonstrated that the classical approach is more labor-intensive than the ligation independent approach (Table 3). The “sequence and ligation independent cloning” (SLIC) system was compared with other conventional expression cloning systems in terms of yield, cost and robustness. The restriction enzyme (RE) based approach was not evaluated in depth since it was obvious from theoretical considerations that the RE approach was too laborious and not well suited for automation in comparison to SLIC cloning. The TOPO cloning system (Invitrogen) was evaluated in addition, but was found to be too imprecise for pool cloning (data not shown). For these reasons we selected SLIC-cloning for the routine high throughput expression cloning workflow.

**Table 3:** Comparison of the classical and the ligation-independent expression cloning workflows.

Classical approach using restriction enzymes and picking ofindividual *E. coli* colonies	Ligation-independent approach avoiding the use of restrictionenzymes and clone picking
Generate and purify PCR fragment	Generate and purify PCR fragment
Prepare vector for ligation	Prepare vector for annealing
Restriction enzyme treatment	T4-DNA polymerase treatment
Purify DNA insert	–
Ligation of fragment with prepared vector	Annealing of fragment with prepared vector
Transformation into competent bacteria	Transformation into competent bacteria
Plate and grow on solid media	Grow in bulk in liquid culture
Pick individual colonies (clones)	–
Grow clones in liquid culture	–
Isolate plasmid DNA from colonies	–
Analyze plasmid DNA from colonies	–
Grow correct clone in liquid culture	–
Isolate plasmid DNA	Isolate plasmid DNA
Transfect eukaryotic cells with expression plasmid	Transfect eukaryotic cells with expression plasmid

In summary, the determination of the threshold for IgG-productivity, the processing of the nucleic acids as a pool avoiding subcloning and the SLIC cloning technology sustain a high overall yield and simplify the B-Cell PCR process thereby making it compatible for automation.

### Synopsis of the Fully Integrated Antibody-generation Process

**Figure 2:**
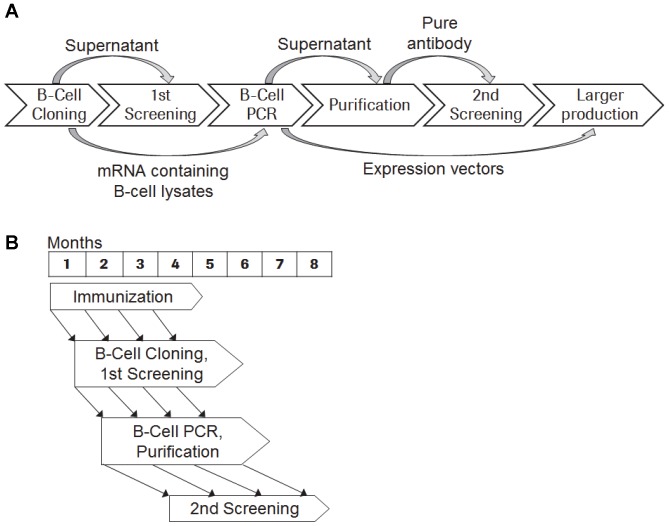
Workflow overviews. (**A**) B-Cell Cloning and B-Cell PCR workflow. (**B**) Schedule depicting the interplay of the different work packages starting with the immunization.

The interplay of all improved steps of the B-Cell Cloning/B-Cell PCR workflow is depicted in Figure 2A. After the initial B-Cell Cloning using peripheral B cells of immunized rabbits, the supernatants of the individual B-cell clones (ASCs) are analyzed by several appropriate biochemical screening assays (primary screening), while the mRNA containing lysates of all B-cell clones are preserved at −20°C. After identification of the supernatants containing the monoclonal antibodies with the desired properties, the corresponding preserved lysates of the B-cell clones are hit-picked and fed into the B-Cell PCR process. After recombinant expression in mammalian cells, the monoclonal rabbit antibodies are purified and thereby concentrated using a chemically defined buffer/medium. The secondary screening round confirms the primary screening and allows additional functional and epitope characterizing assays. After the aforementioned criteria have been satisfied, the expression plasmid of the antibody with the desired properties can be used for a larger scale production. The general time frame is shown in Figure 2B implying that the first B cells were cloned 18 days after the first immunization making this approach extremely fast. All immunizations after the third immunization are conducted at four-week intervals. Each immunization is then followed by the antibody-generation process described above.

### Generation of Monoclonal Rabbit Antibodies Blocking the IL1RL1/IL33-interaction

**Figure 3:**
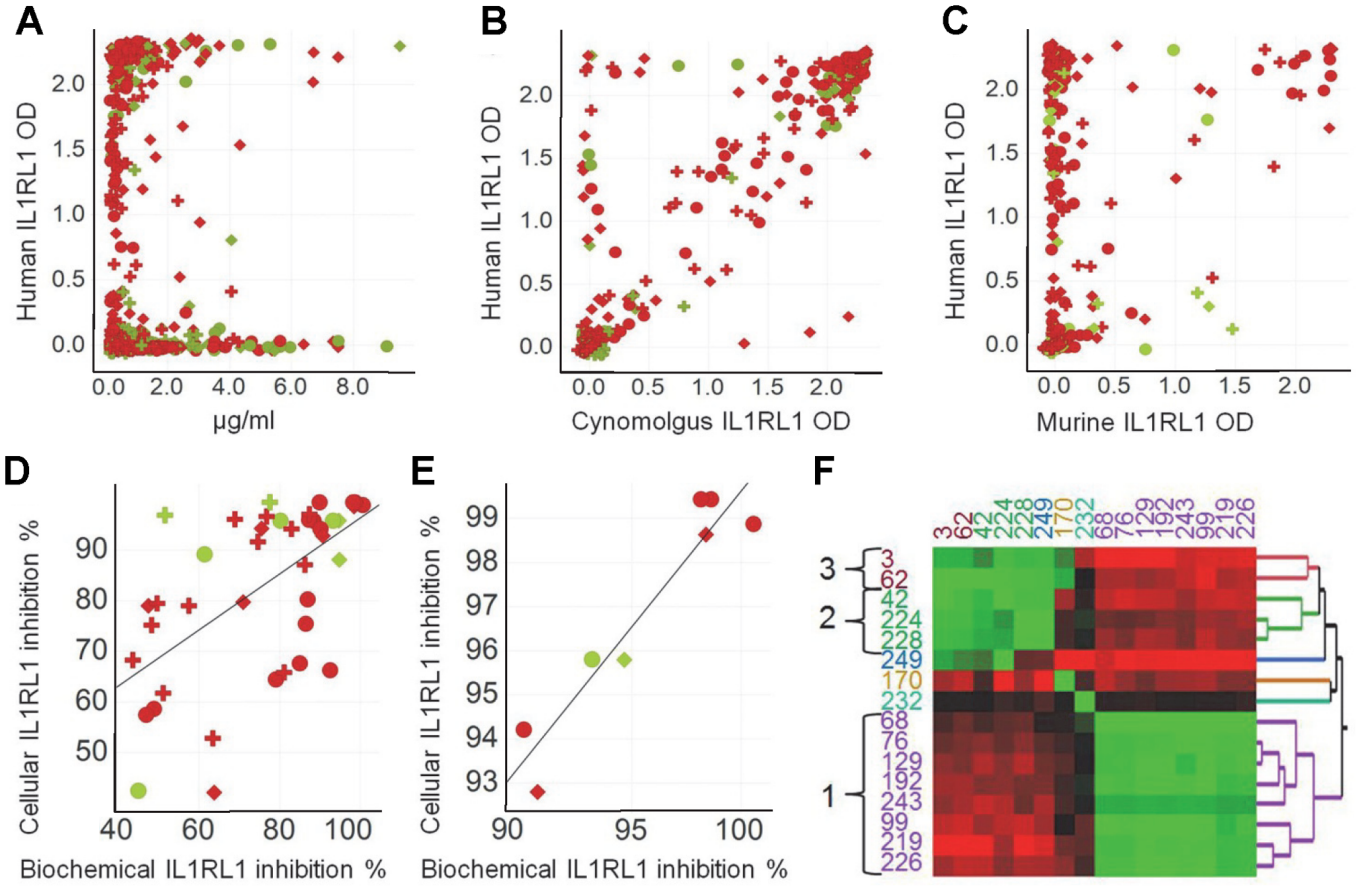
Yield of IL1RL1-specific rabbit antibodies. Scatter Plots depicting the yield of the primary screening using all 7644 supernatants: (**A**) Human IL1RL1 binding (unit: optical density (OD)) versus IgG concentration; (**B**) human IL1RL1 binding versus cynomolgus IL1RL1 binding or versus (**C**) murine IL1RL1 binding. Scatter Plot showing the correlation of the biochemical inhibition assay with the cellular inhibition assay: (**D**) Threshold ≥40% inhibition, RSq: 0.36, (**E**) magnification of Figure 3D using the threshold of >90% inhibition, RSq: 0.9. The statistically confirmed cut off values for the calculation of the percentages were as follows: rabbit IgG-positive >0.013 µg/ml, human IL1RL1-positive >OD 0.195, human Fc-positive ≤OD 0.125, cynomolgus IL1RL1-positive >OD 0.184, murine IL1RL1-positive >OD 0.164. Green are the supernatants deriving from the pre-incubation only scenario and red are the SN after the protein panning step. The diamond, the circle and the cross indicate the three different animals. (**F**) Result of the two dimensional binding matrix identifying different binding epitopes on human IL1RL1. The colored numbers indicate different antigen specific antibodies. The black numbers describe the three antibody groups. The degree of antibody competition in the matrix is depicted by a 3-colour scale with green, black, red color indicating highest competition, mid or lowest competition, respectively.

The optimized B-Cell Cloning and B-Cell PCR workflow was combined to generate inhibitory monoclonal rabbit antibodies to the human IL1RL1 receptor. In total, 7644 individual IgG-positive B cells were deposited from the four blood samples of each of the three IL1RL1 Fc-immunized rabbits. During primary screening all B-cell supernatants were characterized by 6 different biochemical assays. The rabbit IgG production, the binding to the human, cynomolgus and murine IL1RL1 receptors, as well as the blocking capacity of the IL33/IL1RL1 interaction were analyzed. In addition, antibodies against the human IgG1 Fc part of the immunogen were identified and excluded from further analysis. All three immunized rabbits delivered IL1RL1-specific B-cell clones producing IgG over a broad range of concentrations (0.012 µg/ml to >9 µg/ml). The panning on human IL1RL1 protein resulted in elevated numbers of monoclonal IL1RL1-specific antibodies some of them being cross-reactive to cynomolgus and murine IL1RL1 (Figures 3A-C, Table 4). The frequency of IgG producing B-cell clones from the IL1RL1- immunized rabbits (12.8%) was lower than previously seen with the unimmunized rabbits (29% and 44%) potentially due to the more advanced maturation status of the B cells of the immunized animal. But the average IgG concentration of all IgG-secreting B-cell clones was about 0.9 µg/ml which is rather high for ASCs/plasma cells originally derived from single-deposited, non-immortalized peripheral B cells. A counter screen against the human Fc part was necessary since the IL1RL1 antigen for immunization was conjugated to the human Fc part. Of the 978 IgG-positive supernatants, 14.2% bound to the human Fc part and the respective B-cell clones were therefore not further analyzed. The percentage of IgG-positive supernatants of 22.5% binding to the human IL1RL1 protein was significantly higher. Of these, 20.6% were cross-reactive with cynomolgus IL1RL1. Only 5.7% of the IgG-positive supernatants were cross-reactive with murine IL1RL1. More importantly, the IgG concentrations of these supernatants were sufficiently high to biochemically identify antibodies inhibiting the IL33/IL1RL1 interaction. Thus, by using a threshold of 40% inhibition, 16.4% of the antibodies specific for human IL1RL1 functionally blocked the binding of IL33 to its receptor. Among these, 33% of the antibodies showed an inhibition of greater than 80%.

**Table 4:** Results of the primary screening using primary supernatants (SN) of the B-Cell Cloning workflow containing monoclonal rabbit antibodies.

Total Wells	Number of IgG producing B-cell clones (%total wells)	Average IgG concentration of IgG containing wells [µg/ml]	Number of huIL1RL1 binding SN (%IgG-producing B-cell clones)	Number of cynomolgus IL1RL1 binding SN (%IgG-producing B-cell clones)	Number of murine IL1RL1 binding SN (%IgG-producing B-cell clones)	Number of SN being functional in biochemical inhibition assay (%huIL1RL1 binding SN)
7644	978 (12.8)	0.897	220 (22.5)	201 (20.6)	56 (5.7)	36 (16.4)

The statistically confirmed thresholds were rabbit IgG >0.013 µg/ml, human IL1RL1 binding >OD (optical density) 0.195, human Fc binding ≤OD 0.125, cynomolgus IL1RL1 binding >OD 0.184, murine IL1RL1 binding >OD 0.164, and biochemical human IL1RL1:IL33 inhibition ≥40%.

After the primary screening 270 B-cell clones in total were selected for the B-Cell PCR workflow, some of them only partly fulfilling the thresholds depicted in Table 4. After hit-picking, 242 cognate VH and VL pairs were successfully amplified resulting in an amplification efficiency of about 90%. Cloning of the PCR fragments into the respective expression vectors led to 227 plasmid pairs containing the cognate VH or VL inserts. After the antibody production using HEK293 cells this number was confirmed by measuring the rabbit IgG content of the supernatant. Conversely, the remaining 15 incomplete expression vector pairs were negative in this assay. Thus, the SLIC cloning was successful for 94% of the 242 samples thereby proving to be instrumental for maintaining the high overall yield of the DNA cloning procedure. The average IgG productivity after transient co-transfection was 32 µg/ml.

The binding to the human, cynomolgus, and murine IL1RL1 protein as well as the biochemical IL33-blocking capacity of the 227 recombinantly expressed and purified monoclonal rabbit antibodies were confirmed during the secondary screening round. Again 89% of the antibodies showed significant binding to the human IL1RL1 protein and among them 78% were cross-reactive with the cynomolgus IL1RL1 whereas only 18% also bound to the murine IL1RL1 protein. The biochemical ligand binding assay identified 21% of the antibodies as inhibitory for the human IL1RL1/IL33 interaction, and 10% of these antibodies also blocked the murine IL1RL1/IL33 interaction with a threshold of greater than 40%. Among the inhibitory antibodies 8% inhibited both the human and the murine IL1RL1/IL-33 interaction.

We selected 18.5% of the 227 recombinant antibodies showing over 40% inhibition in the biochemical IL1RL1:IL33 interaction assay to be analyzed in an appropriate cellular inhibition assay. Using human IL33-activated NK cells, the selected rabbit antibodies inhibited the IL-33 dependent NK-cell activation with 40% to almost 100% efficacy demonstrating the functionality of these antibodies also at the cellular level. The results of the biochemical blocking assay correlated with the results of the NK-cell dependent inhibition assay at this cut off value (Figure 3D, RSq = 0.36). A stronger correlation could be observed at the higher cut off value of 90% inhibition (Figure 3E, RSq = 0.90) demonstrating that the biochemical inhibition assay as a primary selection criterion was highly predictive for the inhibition of the IL33 binding to the IL1RL1 receptor on the cellular level during secondary screening.

Finally, a cross-competition ELISA was set up to identify IL1RL1-specific antibodies binding to different epitopes, thereby characterizing the diversity of the antibody pool. From the most potent IL33 blocking antibodies, 16 antibodies were assayed against each other and against the other 15 antibodies resulting in a two dimensional binding matrix. The competition data were then analyzed for patterns of competition by the Ward analysis identifying at least 6 major epitope groups with different binding profiles. All antibodies of group 1 were competing with each other but not with the remaining antibodies. Exclusively, antibodies of group 1 were cross-reactive to murine IL1RL1 indicating a distinct epitope clustering (Figure 3F). Group 2 and 3 antibodies competed against each other but showed different competition properties in the presence of antibody 170 and 249. Both antibodies exhibit some asymmetric binding characteristics depending on the preformation of the antibody-antigen complex. This might indicate some differences in affinity or avidity of these antibodies: antibody 170 only competes with group 3 members after pre-incubation with the target, whereas exclusively antibody 232 showed either no or strong competition with any of the other antibodies.

### Sequence Analysis

The clonality and the diversity of the 227 recombinant rabbit antibodies were assessed by analyzing the VH and VL sequences. The analysis of the B-cell clone specific VH and VL sequences confirmed that the single B-cell deposition led to complete monoclonal B-cell clones since only a single VH and VL sequence was identified for each of the 227 antibody-encoding plasmids. This was also confirmed by sequencing the top 20 antibodies after subcloning of the respective *E. coli* clones. Of each of the selected antibodies 5 plasmids were sequenced resulting in a single sequence of each of the antibodies.

**Figure 4:**
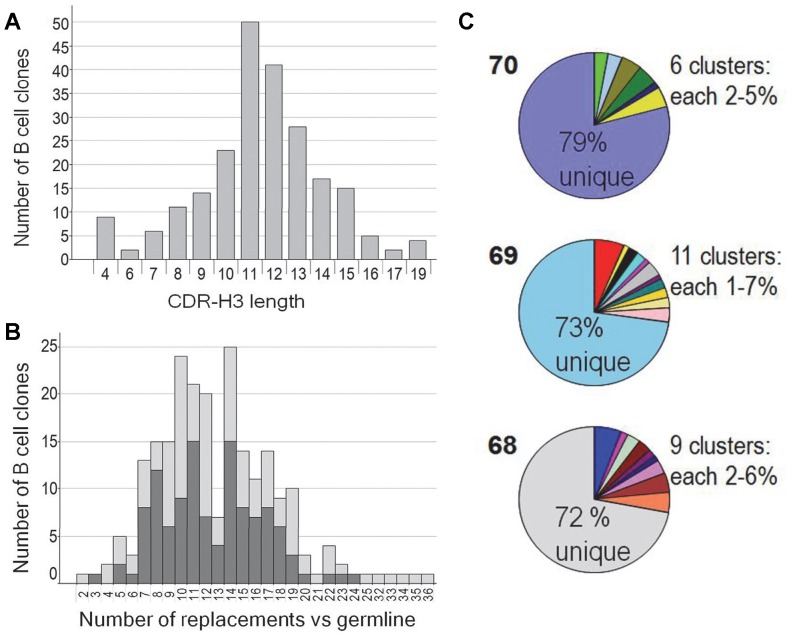
Analysis of the VH-VDJ and VK-VJ sequences to assess clonality and diversity of the recombinant rabbit antibodies. The distribution of the (A) CDR-H3 length (amino acid count) and of the (B) amino acid replacement frequency within the VH region in comparison to VHa1 and VHa3 allotype germ line sequences. Dark grey: VH1a1 germ line gene; light grey: VH1a3 germ line gene. (C) Clustering of the rabbit antibodies according to their CDR-H3 and CDR-L3 sequence similarity. The bold numbers indicate the rabbits.

The diversity of the 227 IL1RL1-binding antibodies was evaluated on the amino acid level by analyzing the CDR-H3 length distribution, the frequency of amino acid replacement mutations in the VHs and the clustering of the CDR-H3 and CDR-L3 sequences of the antibodies. The CDR-H3 length varied between 4 and 19 amino acid residues resulting in a normal Poisson distribution with a mean value of 11.4±2.7 residues (Figure 4A and Table 5) as previously reported for rabbit antibodies isolated by phage display using the spleen of immunized rabbits (11.6±3.1) [3]. The mean values were comparable for the different animals and bleeds (Table 5). In addition, we compared the frequency of amino acid replacement mutations in VHs with the matching VHa1 or VHa3 germ line sequences. The rabbit VH, DH, JH and VL germ line gene sequences used for comparison were collected from different sources [32], [41]–[45]. Per each VH 2 to 36 amino acid replacements were present with a median peak around 10 to 14 and a mean value of 13±5.3 replacements, representing clearly diversified VH regions (Figure 4B and Table 5). Also, in that case no significant difference was observed between animals and bleeds. Finally, we clustered the CDR-H3 and CDR-L3 sequences of the antibodies. Clustering was performed by sequence analysis of CDR3 of VH and VL together. Clustered antibodies are those with identical or very similar CDR-H3 and CDR-L3 sequences including 1 to 2 amino acid replacements. Unique antibodies are those antibodies which CDR3s (VH+VL) are not member of any (VH+VL) CDR3 cluster. Surprisingly, about 75% of the 227 B-cell clones produced unique antibody chains indicating a very high diversity among the IL1RL1-specific peripheral B cells. These unique sequences of the CDR-H3 and CDR-L3 gene segments did not only differ between the individual B-cell clones of one rabbit but were also different between the three rabbits.

**Table 5:** Functionality and sequence diversity of the recombinant antibodies per animals and bleeds.

Bleed	Animal	Number of recombinant rabbit antibodies	Number of recombinant and functional* rabbit antibodies	Number of somatic amino acid replacements in VH			CDR-H3 length			Unique VH+VL	% Unique VH+VL per animal and bleed	Clustered VH+VL	Identical CDR-H3	Identical CDR-L3	Identical CDR-H3+CDR-L3	Identical VHs	Identical VLs	Identical VH+VL
				Range	Average	STDEV	Range	Average	STDEV									
1	68	30	2	4–22	12,6	6,1	7–19	11,5	2,4	23	76	7	4	4	3	0	2	0
1	69	42	7	7–19	11,6	3,3	4–15	11	2,2	33	78	10	4	5	0	0	0	0
1	70	17	2	8–25	13,1	5,1	10–16	12,2	1,6	14	82	4	0	4	0	0	0	0
2	68	30	3	2–34	13	6,4	4–16	11,1	3,5	21	70	9	5	6	2	0	0	0
2	69	21	5	5–36	14	8,2	4–16	11,5	2,7	15	71	6	4	3	4	0	2	0
2	70	26	4	3–19	12,3	3,7	4–19	11,9	3,8	20	77	7	4	2	0	0	0	0
3	68	6	1	4–22	14,1	5,7	9–12	11	1,1	3	50	3	0	1	0	0	0	0
3	69	24	6	9–35	15,6	5	6–16	11,1	2,6	17	70	8	6	1	0	0	0	0
3	70	10	5	8–16	10,7	3	10–15	12,2	1,9	8	80	2	1	0	0	0	0	0
4	68	2	2	14;17	nd	nd	8;13	nd	nd	2	100	0	0	0	0	0	0	0
4	69	5	1	9–19	15,2	3,7	4–12	9,8	3,2	2	40	3	2	2	2	2	2	2
4	70	14	6	7–22	13,8	4,8	7–17	11,9	2,9	11	78	3	0	1	0	0	0	0

* ”Functional” refers to the biochemical IL1RL1:IL33 inhibition assay with a cut off of ≥40% inhibition.

Only around 25% of the cognate VH and VL pairs could be clustered in 6 to 11 sequence families of 2 to 7 B-cell clones per family. None of the VH/VL clusters of one animal was found in any of the other two animals implying that each animal delivered distinct clusters of B-cell clones (Figure 4C and Table 5), whereas, in some cases (2 clusters for animal #68, 6 for animal #69 and 4 for animal #70) members of a cluster were found in different bleeds. Even in these clusters an increase of replacement mutations over time was not observed (data not shown). Taken together, the diversity is mainly triggered by animal heterogeneity.

The 16 antibodies belonging to the 6 different epitopes as identified by the Ward analysis (Figure 3F) did not show any particular sequence similarity or shared sequence features. Furthermore, the sequence clustering using the CDR3s of the cognate VH/VL and the functional properties of the antibodies could not be correlated in a statistically significant way with the epitope cluster.

## Discussion

We have established a robust and efficient high throughput semi-automated platform for the generation of monoclonal antibodies from peripheral rabbit B cells. The proof-of-concept study using IL1RL1-immunized rabbits demonstrated that the combination of the B-Cell Cloning and B-Cell PCR technology is highly effective in isolating a large number of rabbit B-cell clones secreting sufficient monoclonal antigen specific IgG. Already during the primary screening using 6 different assays the unambiguous screening results reliably identified several antibodies that were cross-reactive to cynomolgus and murine IL1RL1 as well as inhibitory for the IL33:IL1RL1 interaction. On the basis of this finding we strive to maintain or even increase the IgG content in the supernatant of the primary B-cell clone allowing a comprehensive primary screening also for future immunization campaigns. By use of the technology described here, the workload and the costs of the subsequent B-cell PCR workflow will be significantly reduced, because exclusively the most promising antibody candidates from the primary screening will be processed.

In addition, the simplified and semi-automated B-Cell PCR workflow maintained a high yield of the selected antibodies throughout the multitude of consecutive molecular biology steps mainly for two reasons. First, by ensuring the monoclonality through the single cell sorting step, the genetic information of the antibody producing B-cell clone could be used as a pool of nucleic acids. Second, the expression cloning was greatly facilitated by the introduction of the SLIC cloning method avoiding the use of restriction enzymes and revealing the tremendously high efficiency of 94%. Both facts together enable the circumvention of the laborious single colony picking of *E. coli* colonies representing the main obstacle for automation. In contrast, methods starting with oligo- or polyclonal B-cells [16], [17] cannot fulfill the requirement of complete monoclonality implying the need for tedious efforts to identify the cognate VH- and VL pairs among a pool of several possible HCs and LCs.

The sequence diversity of the antigen specific rabbit antibodies obtained from blood was very high, in accordance with published literature describing the sequence diversity of peripheral human B cells [15]. One explanation for the large diversity could be that only a fraction of the antigen-specific B-cell clones has been recruited by the workflow described herein although about one quarter of the 978 IgG-secreting B-cell clones were found to bind the antigen. On the other hand, peripheral B cells could be a preferred source for very good antibodies in terms of affinity maturation, since the emigration of the matured ASCs/plasma blasts from the spleen is an affinity driven process [15], [46], [47].

Potential areas of further optimization of the entire technology have already been identified. Since the frequency of the IgG-secreting B-cell clones of 13% is not very high as shown by the data presented in the proof-of-concept study, we seek to further increase this frequency by optimizing the single cell sort and the cultivation conditions leading to more and higher producing rabbit B-cell clones. The use of synthetic media and tailored cytokine cocktails for the B-cell cultivation would facilitate the analysis of secreted antibodies in functional assays. The humanization of rabbit antibodies as a prerequisite for therapeutic antibodies has already been demonstrated [5], but progress on this topic is still ongoing. However, in comparison to already existing technologies such as hybridoma generation and phage display approaches using immunized animals we see some drawbacks: the need of an expensive single cell sorting device being an integral part of this technology, and the limited amounts of the primary B-cell supernatants that require the development of very sensitive primary screening assays.

Besides the sequence analysis of one immunization campaign presented here, a thorough analysis of the temporal and spatial development of the immune repertoire on a statistically valid basis could be performed by a quantitative and qualitative comparison of the antigen specific antibodies from each of the four time points of blood sampling or from different immunological organs (blood, lymph nodes, spleen) using the large data set obtained from several immunization campaigns with different antigens.

Apart from the obvious time saving this technology offers at least three important additional advantages. First, the animals don’t need to be sacrificed in order to obtain the antibodies which leads to the following options: (i) the immunization protocol could be continued at any time, e.g. for the generation of polyclonal antibodies, (ii) it is possible to monitor the B-cell response and B-cell repertoire during the immunization campaign and adapt the immunization protocol if required, and (iii) animals (e.g. transgenic rabbits) can be returned to breeding after an immunization campaign. Second, the genetic information of the antigen specific monoclonal antibody is preserved at a very early time point during the antibody generation process thereby temporally uncoupling the screening from the cell cultivation, which is in contrast to the antibody generation by hybridoma fusions. Finally, the availability of the sequence of the antigen specific rabbit antibody at an early time point (i) facilitates the hit selection by identifying doublets and antibody clusters, (ii) provides information on the diversity and (iii) accelerates the generation of other antibody formats (e.g. isotype switched or bispecific antibodies).

In summary, the combination of the B-Cell Cloning and B-Cell PCR technology presented here represents a powerful state of the art procedure for obtaining antigen specific and functional monoclonal rabbit antibodies from peripheral blood.

## Supporting Information

Figure S1 **Plasmid maps. (A)** Circular plasmid map of the ‘Rabbit IgG HC Vector’ showing all elements mentioned in the Materials and Methods section. The vector is linearized by either *Bam*HI or *Xba*I restriction sites positioned side by side. **(B)** Circular plasmid map of the 'Rabbit kappa LC Vector' showing all elements mentioned in the Materials and Methods section. After linearization by *Apa*I and *Eco*RI restriction sites the V-kappa and a part of the C-kappa sequence is deleted.
(TIFF)

## References

[1] Zhu W, Yu G (2009) Rabbit Hybridoma. In: An Z, editors. Therapeutic Monoclonal Antibodies: From Bench to Clinic. John Wiley & Sons, Inc. pp. 151–168.

[2] Spieker-Polet H, Sethupathi P, Yam PC, Knight KL (1995) Rabbit monoclonal antibodies: generating a fusion partner to produce rabbit-rabbit hybridomas. Proc Natl Acad Sci U S A 92: 9348–9352. 7568130 10.1073/pnas.92.20.9348 PMC40982

[3] Popkov M, Mage RG, Alexander CB, Thundivalappil S, Barbas CF, et al (2003) Rabbit immune repertoires as sources for therapeutic monoclonal antibodies: the impact of kappa allotype-correlated variation in cysteine content on antibody libraries selected by phage display. J Mol Biol 325: 325–335. 12488098 10.1016/s0022-2836(02)01232-9

[4] Rader C, Ritter G, Nathan S, Elia M, Gout I, et al (2000) The rabbit antibody repertoire as a novel source for the generation of therapeutic human antibodies. J Biol Chem 275: 13668–13676. 10788485 10.1074/jbc.275.18.13668

[5] Rief N, Waschow C, Nastainczyk W, Montenarh M, Gotz C (1998) Production and characterization of a rabbit monoclonal antibody against human CDC25C phosphatase. Hybridoma 17: 389–394. 9790074 10.1089/hyb.1998.17.389

[6] Zubler RH, Erard F, Lees RK, Van LM, Mingari C, et al (1985) Mutant EL-4 thymoma cells polyclonally activate murine and human B cells via direct cell interaction. J Immunol 134: 3662–3668. 3886789

[7] Babcook JS, Leslie KB, Olsen OA, Salmon RA, Schrader JW (1996) A novel strategy for generating monoclonal antibodies from single, isolated lymphocytes producing antibodies of defined specificities. Proc Natl Acad Sci U S A 93: 7843–7848. 8755564 10.1073/pnas.93.15.7843 PMC38836

[8] Corti D, Voss J, Gamblin SJ, Codoni G, Macagno A, et al (2011) A neutralizing antibody selected from plasma cells that binds to group 1 and group 2 influenza A hemagglutinins. Science 333: 850–856. 21798894 10.1126/science.1205669

[9] Dohmen SE, Mulder A, Verhagen OJ, Eijsink C, Franke-van Dijk ME, et al (2005) Production of recombinant Ig molecules from antigen-selected single B cells and restricted usage of Ig-gene segments by anti-D antibodies. J Immunol Methods 298: 9–20 10.1016/j.jim.2004.12.013. 15847793 10.1016/j.jim.2004.12.013

[10] Jin A, Ozawa T, Tajiri K, Obata T, Kondo S, et al (2009) A rapid and efficient single-cell manipulation method for screening antigen-specific antibody-secreting cells from human peripheral blood. Nat Med 15: 1088–1092. 19684583 10.1038/nm.1966

[11] Meijer PJ, Andersen PS, Haahr HM, Steinaa L, Jensen A, et al (2006) Isolation of human antibody repertoires with preservation of the natural heavy and light chain pairing. J Mol Biol 358: 764–772. 16563430 10.1016/j.jmb.2006.02.040

[12] Scheid JF, Mouquet H, Ueberheide B, Diskin R, Klein F, et al (2011) Sequence and structural convergence of broad and potent HIV antibodies that mimic CD4 binding. Science 333: 1633–1637. 21764753 10.1126/science.1207227 PMC3351836

[13] Tiller T, Meffre E, Yurasov S, Tsuiji M, Nussenzweig MC, et al (2008) Efficient generation of monoclonal antibodies from single human B cells by single cell RT-PCR and expression vector cloning. J Immunol Methods 329: 112–124 10.1016/j.jim.2007.09.017. 17996249 10.1016/j.jim.2007.09.017 PMC2243222

[14] Weitkamp JH, Kallewaard N, Kusuhara K, Feigelstock D, Feng N, et al.. (2003) Generation of recombinant human monoclonal antibodies to rotavirus from single antigen-specific B cells selected with fluorescent virus-like particles. J Immunol Methods 275: 223–237. S0022175903000139 [pii]. 10.1016/s0022-1759(03)00013-9 12667686

[15] Wrammert J, Smith K, Miller J, Langley WA, Kokko K, et al (2008) Rapid cloning of high-affinity human monoclonal antibodies against influenza virus. Nature 453: 667–671. 18449194 10.1038/nature06890 PMC2515609

[16] Lightwood DJ, Carrington B, Henry AJ, McKnight AJ, Crook K, et al (2006) Antibody generation through B cell panning on antigen followed by in situ culture and direct RT-PCR on cells harvested en masse from antigen-positive wells. J Immunol Methods 316: 133–143. 17027850 10.1016/j.jim.2006.08.010

[17] Weber M, Weiss E, Engel AM (2003) Combining EL4-B5-based B-cell stimulation and phage display technology for the successful isolation of human anti-Scl-70 autoantibody fragments. J Immunol Methods 278: 249–259. S002217590300228X [pii]. 10.1016/s0022-1759(03)00228-x 12957412

[18] Lee JH, Wang LC, Yu HH, Lin YT, Yang YH, et al (2010) Type I IL-1 receptor (IL-1RI) as potential new therapeutic target for bronchial asthma. Mediators Inflamm 2010: 567351. 10.1155/2010/567351 PMC2910497 20671916

[19] Oboki K, Ohno T, Kajiwara N, Saito H, Nakae S (2010) IL-33 and IL-33 receptors in host defense and diseases. Allergol Int 59: 143–160. 20414050 10.2332/allergolint.10-RAI-0186

[20] Pastorelli L, Garg RR, Hoang SB, Spina L, Mattioli B, et al (2010) Epithelial-derived IL-33 and its receptor ST2 are dysregulated in ulcerative colitis and in experimental Th1/Th2 driven enteritis. Proc Natl Acad Sci U S A 107: 8017–8022. 20385815 10.1073/pnas.0912678107 PMC2867895

[21] Palmer G, Talabot-Ayer D, Lamacchia C, Toy D, Seemayer CA, et al (2009) Inhibition of interleukin-33 signaling attenuates the severity of experimental arthritis. Arthritis Rheum 60: 738–749. 19248109 10.1002/art.24305

[22] Moussion C, Ortega N, Girard JP (2008) The IL-1-like cytokine IL-33 is constitutively expressed in the nucleus of endothelial cells and epithelial cells in vivo: a novel ‘alarmin’? PLoS One 3: e3331. 10.1371/journal.pone.0003331 PMC2556082 18836528

[23] Tominaga S, Yokota T, Yanagisawa K, Tsukamoto T, Takagi T, et al (1992) Nucleotide sequence of a complementary DNA for human ST2. Biochim Biophys Acta 1171: 215–218. 1482686 10.1016/0167-4781(92)90125-j

[24] Ali S, Huber M, Kollewe C, Bischoff SC, Falk W, et al (2007) IL-1 receptor accessory protein is essential for IL-33-induced activation of T lymphocytes and mast cells. Proc Natl Acad Sci U S A 104: 18660–18665. 18003919 10.1073/pnas.0705939104 PMC2141833

[25] Coyle AJ, Lloyd C, Tian J, Nguyen T, Erikkson C, et al (1999) Crucial role of the interleukin 1 receptor family member T1/ST2 in T helper cell type 2-mediated lung mucosal immune responses. J Exp Med 190: 895–902. 10510079 10.1084/jem.190.7.895 PMC2195643

[26] Steenbakkers PG, Hubers HA, Rijnders AW (1994) Efficient generation of monoclonal antibodies from preselected antigen-specific B cells. Efficient immortalization of preselected B cells. Mol Biol Rep 19: 125–134. 8072493 10.1007/BF00997158

[27] Becker RS, Knight KL (1990) Somatic diversification of immunoglobulin heavy chain VDJ genes: evidence for somatic gene conversion in rabbits. Cell 63: 987–997. 2124176 10.1016/0092-8674(90)90502-6

[28] Mage RG, Sehgal D, Schiaffella E, Anderson AO (1999) Gene-conversion in rabbit B-cell ontogeny and during immune responses in splenic germinal centers. Vet Immunol Immunopathol 72: 7–15. 10614487 10.1016/s0165-2427(99)00110-5

[29] Schiaffella E, Sehgal D, Anderson AO, Mage RG (1999) Gene conversion and hypermutation during diversification of VH sequences in developing splenic germinal centers of immunized rabbits. J Immunol 162: 3984–3995. 10201919

[30] Tunyaplin C, Knight KL (1995) Fetal VDJ gene repertoire in rabbit: evidence for preferential rearrangement of VH1. Eur J Immunol 25: 2583–2587. 7589130 10.1002/eji.1830250927

[31] Lanning D, Zhu X, Zhai SK, Knight KL (2000) Development of the antibody repertoire in rabbit: gut-associated lymphoid tissue, microbes, and selection. Immunol Rev 175: 214–228. 10933605

[32] Ros F, Reichenberger N, Dragicevic T, van Schooten WC, Buelow R, et al (2005) Sequence analysis of 0.4 megabases of the rabbit germline immunoglobulin kappa1 light chain locus. Anim Genet 36: 51–57. 15670131 10.1111/j.1365-2052.2004.01221.x

[33] Kozak M (1987) An analysis of 5′-noncoding sequences from 699 vertebrate messenger RNAs. Nucleic Acids Res 15: 8125–8148. 3313277 10.1093/nar/15.20.8125 PMC306349

[34] Haun RS, Serventi IM, Moss J (1992) Rapid, reliable ligation-independent cloning of PCR products using modified plasmid vectors. Biotechniques 13: 515–518. 1362067

[35] Li MZ, Elledge SJ (2007) Harnessing homologous recombination in vitro to generate recombinant DNA via SLIC. Nat Methods 4: 251–256. 17293868 10.1038/nmeth1010

[36] Smithgall MD, Comeau MR, Yoon BR, Kaufman D, Armitage R, et al (2008) IL-33 amplifies both Th1- and Th2-type responses through its activity on human basophils, allergen-reactive Th2 cells, iNKT and NK cells. Int Immunol 20: 1019–1030. 18550585 10.1093/intimm/dxn060

[37] Ward JH Jr (1963) Hierarchical Grouping to Optimize an Objective Function. Journal of the American Statistical Association 58: 236–244.

[38] Johnson NL (1949) Systems of frequency curves generated by methods of translation. Biometrika 36: 149–176. 18132090

[39] Kehrl JH, Muraguchi A, Fauci AS (1984) Human B cell activation and cell cycle progression: stimulation with anti-mu and Staphylococcus aureus Cowan strain I. Eur J Immunol. 14: 115–121. 10.1002/eji.1830140203 6199210

[40] Yoshimura A, Lien E, Ingalls RR, Tuomanen E, Dziarski R, et al (1999) Cutting edge: recognition of Gram-positive bacterial cell wall components by the innate immune system occurs via Toll-like receptor 2. J Immunol 163: 1–5. 10384090

[41] Chen HT, Alexander CB, Chen FF, Mage RG (1996) Rabbit DQ52 and DH gene expression in early B-cell development. Mol Immunol 33: 1313–1321. 9171891 10.1016/s0161-5890(96)00107-1

[42] Knight KL, Becker RS (1990) Molecular basis of the allelic inheritance of rabbit immunoglobulin VH allotypes: implications for the generation of antibody diversity. Cell 60: 963–970. 2317867 10.1016/0092-8674(90)90344-e

[43] Sehgal D, Johnson G, Wu TT, Mage RG (1999) Generation of the primary antibody repertoire in rabbits: expression of a diverse set of Igk-V genes may compensate for limited combinatorial diversity at the heavy chain locus. Immunogenetics 50: 31–42. 10541804 10.1007/s002510050683

[44] Hole NJ, Young-Cooper GO, Mage RG (1991) Mapping of the duplicated rabbit immunoglobulin kappa light chain locus. Eur J Immunol 21: 403–409. 1671838 10.1002/eji.1830210223

[45] Ros F, Puels J, Reichenberger N, van Schooten W, Buelow R, et al (2004) Sequence analysis of 0.5 Mb of the rabbit germline immunoglobulin heavy chain locus. Gene 330: 49–59. 15087123 10.1016/j.gene.2003.12.037

[46] Paus D, Phan TG, Chan TD, Gardam S, Basten A, et al (2006) Antigen recognition strength regulates the choice between extrafollicular plasma cell and germinal center B cell differentiation. J Exp Med 203: 1081–1091. 16606676 10.1084/jem.20060087 PMC2118299

[47] Smith KG, Light A, Nossal GJ, Tarlinton DM (1997) The extent of affinity maturation differs between the memory and antibody-forming cell compartments in the primary immune response. EMBO J 16: 2996–3006. 9214617 10.1093/emboj/16.11.2996 PMC1169918

