# Supplementary figures and images for: A Robust High Throughput Platform to Generate Functional Recombinant Monoclonal Antibodies Using Rabbit B Cells from Peripheral Blood
Source: PLoS One. 2014 Feb 4;9(2):e86184. doi: 10.1371/journal.pone.0086184 (PMC3913575; doi:10.1371/journal.pone.0086184)

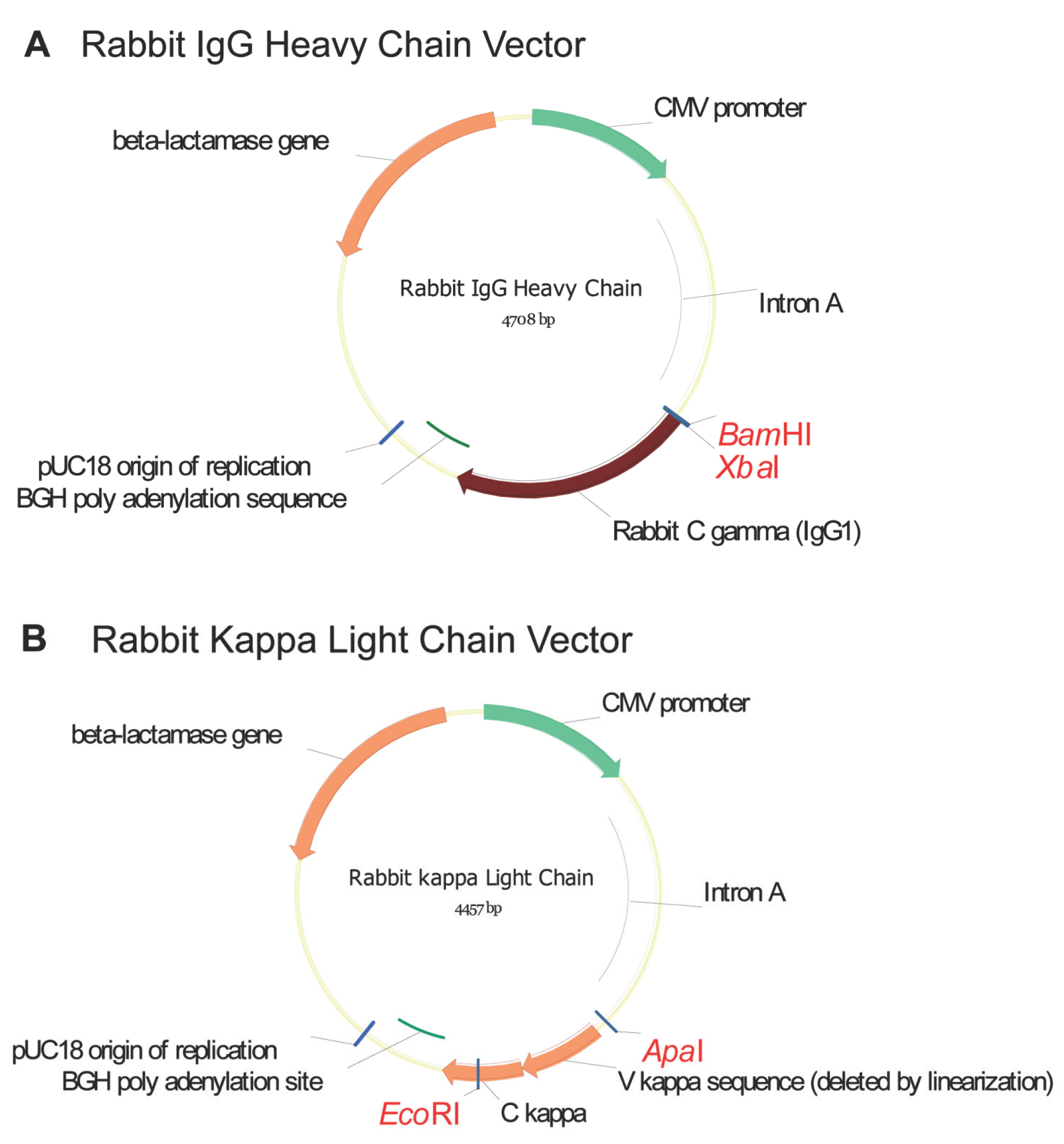

Supplement: Figure S1 — Plasmid maps. (A) Circular plasmid map of the ‘Rabbit IgG HC Vector’ showing all elements mentioned in the Materials and Methods section. The vector is linearized by either BamHI or XbaI restriction sites positioned side by side. (B) Circular plasmid map of the 'Rabbit kappa LC Vector' showing all elements mentioned in the Materials and Methods section. After linearization by ApaI and EcoRI restriction sites the V-kappa and a part of the C-kappa sequence is deleted. (TIFF) [file pone.0086184.s001.tiff]
